# Immunity to HIV in Early Life

**DOI:** 10.3389/fimmu.2014.00391

**Published:** 2014-08-12

**Authors:** Maximilian Muenchhoff, Andrew J. Prendergast, Philip Jeremy Renshaw Goulder

**Affiliations:** ^1^Department of Paediatrics, University of Oxford, Peter Medawar Building for Pathogen Research, Oxford, UK; ^2^Centre for Paediatrics, Blizard Institute, Queen Mary University of London, London, UK; ^3^Zvitambo Institute for Maternal and Child Health Research, Harare, Zimbabwe; ^4^HIV Pathogenesis Programme, Doris Duke Medical Research Institute, Nelson R. Mandela School of Medicine, University of KwaZulu-Natal, Durban, South Africa

**Keywords:** HIV, pediatric, innate immunity, adaptive immunity, immune responses, immune activation, immune exhaustion, viral reservoir

## Abstract

The developing immune system is adapted to the exposure to a plethora of pathogenic and non-pathogenic antigens encountered *in utero* and after birth, requiring a fine balance between protective immunity and immune tolerance. In early stages of life, this tolerogenic state of the innate and adaptive immune system and the lack of immunological memory render the host more susceptible to infectious pathogens like HIV. HIV pathogenesis is different in children, compared to adults, with more rapid disease progression and a substantial lack of control of viremia compared to adults. Plasma viral load remains high during infancy and only declines gradually over several years in line with immune maturation, even in rare cases where children maintain normal CD4 T-lymphocyte counts for several years without antiretroviral therapy (ART). These pediatric slow progressors also typically show low levels of immune activation despite persistently high viremia, resembling the phenotype of natural hosts of SIV infection. The lack of immunological memory places the fetus and the newborn at higher risk of infections; however, it may also provide an opportunity for unique interventions. Frequencies of central memory CD4+ T-lymphocytes, one of the main cellular reservoirs of HIV, are very low in the newborn child, so immediate ART could prevent the establishment of persistent viral reservoirs and result in “functional cure.” However, as recently demonstrated in the case report of the “Mississippi child” who experienced viral rebound after more than 2 years off ART, additional immunomodulatory strategies might be required for sustained viral suppression after ART cessation. In this review, we discuss the interactions between HIV and the developing immune system in children and the potential implications for therapeutic and prophylactic interventions.

## Introduction

**Figure 1:**
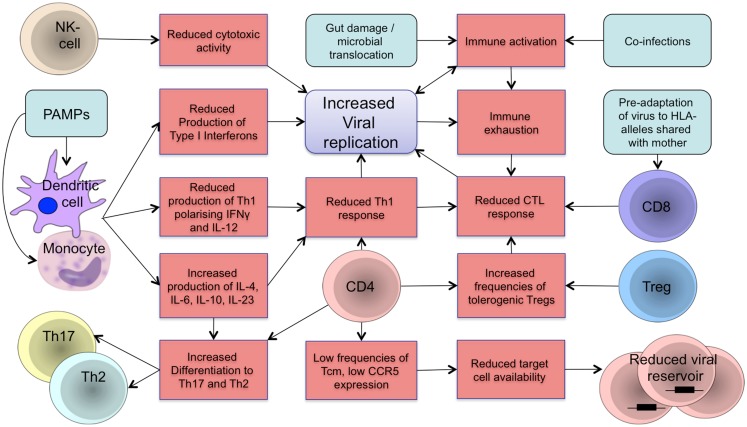
**Schematic representation of components of the developing immune system in HIV infection**. The developing immune system is characterized by an attenuated pro-inflammatory innate immune response with a shift toward Th2/Th17 polarizing cytokines to protect the organism against extracellular pathogens and reduce the risk of autoimmunity and inflammation. A tolerogenic state of the immune system is supported by increased frequencies of regulatory T-cells that suppress the activity of CD4 and CD8 T-cells. Microbial translocation and viral replication result in immune activation and immune exhaustion that lead to immune dysfunction and loss of immune control resulting in further viral replication in a positive feedback loop. Differences in target cell availability and memory differentiation in early life affect the size and composition of the viral reservoir in children. Main aspects of the developing immune system in regards to vertical HIV infection are summarized. Treg: regulatory T-cells; NK cell: natural killer cell; PAMPs: pathogen-associated molecular patterns; CTL: cytotoxic T-lymphocyte.

In the dynamic developmental period from fetal life to adolescence, the human organism is more susceptible and vulnerable to chronic viral infections (1). HIV pathogenesis in early life is characterized by faster disease progression and shorter time to AIDS and death, compared to adults. The innate and adaptive arms of the immune system are more tolerogenic soon after birth and fail to control viral replication in infancy. Persistent viremia, reactivation of co-infections, and microbial translocation during chronic infection drive HIV pathogenesis through increased immune activation, resulting in immune dysregulation and functional immune exhaustion that exacerbate disease progression in a positive feedback loop (2–4) as visualized in Figure 1.

However, the recent report of a child who apparently achieved “functional cure” for more than 2 years following early therapeutic intervention (5) has raised major interest in the development of therapeutic strategies that could potentially be applied for cure even beyond the pediatric setting. In this review, we will discuss immunity to HIV in early life and how it affects pathogenesis and disease outcome, but also offers unique opportunities for prophylactic and therapeutic interventions in pediatric HIV infection.

## Pediatric HIV Infection: Epidemiology

Of the estimated 35 million people living with HIV today, 3.3 million are children (UNAIDS 2013 report). Administration of combination antiretroviral therapy (ART) can reduce risk of transmission to approximately 1–2% (6) and WHO now recommends the use of triple-drug ART regimens in all pregnant and breastfeeding women regardless of CD4 count, either lifelong (formerly referred to as option B+) or only during pregnancy and breastfeeding (formerly referred to as option B) (7). Despite increasing availability of ART, many women are diagnosed with HIV late in pregnancy and prevention of mother-to-child transmission (PMTCT) coverage rates remain suboptimal in many countries, resulting in 260,000 children becoming newly infected with HIV in 2012.

## Vertical HIV Transmission

Without PMTCT, the overall risk of HIV transmission is up to 40% in sub-Saharan Africa; infection can occur *in utero* (5–10%), intrapartum (15%), or post-partum via breastfeeding (15%) (8). Interestingly, MTCT rates are much lower (<7%) in the natural hosts of SIV infection (such as sooty mangabeys), compared to humans (30–40%) or rhesus macaques (25–75%) (9). During breastfeeding, no transmissions have been observed in sooty mangabeys, despite high plasma and breast milk viral loads (10). This has been attributed to paucity of CCR5+ CD4+ target cells for SIV infection (11), since the vast majority of vertically transmitted SIV/HIV-strains are R5-tropic and therefore depend on CCR5 as a co-receptor to infect CD4+ T-cells (12). Indeed, a recent study has shown very low to absent populations of CCR5+ CD4+ T-cells in the peripheral blood compartment and especially in the intestinal tract of sooty mangabeys compared to rhesus macaques (13). In human infants, CCR5+ CD4+ T-cells are virtually absent in cord blood (14). However, CCR5+ CD4+ T-cells are abundant in the intestinal mucosa and these cells are highly susceptible to HIV infection (15). The increased availability of target cells in the intestinal tract, in combination with the effectiveness of the placental barrier prior to labor, are both likely to contribute to the higher risk of intra- and post-partum, compared to *in utero*, transmission.

## Clinical Course

Once infection is established, the course of disease progression in vertically infected infants is generally faster than in adults, with a rapid decline of CD4 cells and onset of recurrent infections, failure to thrive, and delayed neurodevelopment. In adult HIV infection, survival times decrease with increasing age at seroconversion, with a median time to AIDS and death of approximately a decade without ART (16). In contrast, mortality among vertically infected children in Africa exceeds 50% by the age of 2 years in the absence of ART (17). However, precise timing of transmission is an important determinant of survival: in a recent meta-analysis, 1-year mortality was 26% among children infected postnatally through breastfeeding, compared to 52% if infected perinatally (18). Another study that distinguished *in utero*, intrapartum and postnatal infection found median survival times from infection of 208, 380, and >500 days, respectively (19). Furthermore, lower mortality rates have been observed that the later HIV is acquired postnatally via breastfeeding (20). Interestingly, a cohort of children with hemophilia, who acquired HIV infection between the age of 5 and 14 years, had significantly longer AIDS-free survival times than adults (16). Long-term survival rates in vertically infected untreated children are estimated to be higher than initially appreciated with a probability of 20–30% surviving up to 10 years if infected perinatally and 16 years if infected via breastfeeding (20, 21).

Absolute CD4 count and viral load levels, which are well established clinical markers of disease progression in adults, have to be interpreted differently in children. The absolute CD4 count is three- to fourfold higher in the newborn compared to adults and gradually declines with age to reach adult levels after about 6 years or later (22, 23). The percentage of CD4+ T-cells is less variable with age and often used as an indicator of CD4 depletion in young children. HIV-1 plasma RNA levels are generally higher in vertically infected children than in adults and peak viremia (in the region of 5–7 log copies per milliliter) is observed around 3 months of age (24). Peak viremia is higher in children infected intrapartum than *in utero* (25) and lower in postnatally infected children, suggesting improved viral control by the more mature immune system (26, 27). Whereas in adult infection, a steady state viral set point is reached within several weeks of infection (28, 29), viremia persists at high levels, and only declines slowly until a quasi-set point is reached after several years in children who survive (30). This gradual control of viral replication has been associated with increasing maturation and development of the immune system (1).

## Innate Immune System

In the initial response to infections, the innate immune system performs two broad roles. On the one hand, it directly inactivates or attenuates invading pathogens, and on the other hand, it instructs the adaptive arm of the immune system to generate appropriate effector responses. Innate immune cells, such as macrophages, monocytes, and dendritic cells interact with pathogen-associated molecular patterns (PAMPs) through pattern recognition receptors (PRRs) that have evolved as substrate-specific receptors that can discriminate between self- and non-self and constantly sample the intra- and extracellular milieu [reviewed in Ref. (31)]. PRRs include the Toll-like receptors (TLRs), which are mainly located within the membranes of the cell surface and endosomes, and intracellular sensors such as RIG-1 like receptors (RLRs). HIV is principally detected by the innate immune system through the recognition of viral nucleic acids that are present in the cytosol of productively infected cells during the viral replication cycle and in the endosomes of phagocytosing cells. For example, cell-free virus that is phagocytosed by dendritic cells is recognized in the endosome by TLR-7 and TLR-9, which bind single-stranded RNA and DNA, respectively. Most PAMP/PRR interactions activate signaling pathways that converge in transcriptional activation of pro-inflammatory cytokines and type-1 interferons (IFNs). Type 1 IFNs bind to receptors of infected and neighboring cells and induce expression of IFN-stimulated genes (ISGs) that synergistically inhibit viral replication and spread (32).

The importance of these innate immune mechanisms in viral infections has been demonstrated in children with gene mutations in the TLR3 signaling pathway, who are prone to severe herpes simplex encephalitis (33). Certain polymorphisms in the TLR-9 gene were associated with increased risk of mother-to-child transmission of HIV in one study (34), but further independent studies on innate immune signaling in pediatric HIV infection are required. Genetic polymorphisms of APOBEC3G, a host restriction factor that is induced by type-1 IFNs and directly inhibits replication of HIV, are associated with increased viral loads (35), lower CD4 counts (36), and more rapid disease progression in HIV-infected children (37).

Antigen-presenting innate immune cells such as dendritic cells play a key role not only in inducing pathways that directly inhibit viral replication but also in regulating the host adaptive immune response to HIV infection. Upon TLR stimulation, neonatal dendritic cells and monocytes are less polyfunctional and produce less type-1 IFNs than adult cells (38). However, it has been noted that neonatal innate immune cells do not have a generalized deficit in secretion of pro-inflammatory cytokines, but rather display distinct cytokine profiles to shape differential adaptive immune responses compared to adults. The most consistently reported difference in neonatal innate immune cell cytokine patterns is a relatively low production of Th1-polarizing cytokines, such as Interleukin-12 and IFN-gamma, in favor of Th2- and Th17-inducing Interleukin-10, Interleukin-6, and Interleukin-23 (38, 39) [reviewed in Ref. (40)]. This immunoregulatory strategy has likely evolved for two reasons: first, to dampen pro-inflammatory Th1 responses that could be damaging *in utero* and after birth when the newborn is exposed to an abundance of newly encountered antigens; and second, to ensure protection against extracellular bacterial and fungal pathogens, which can be rapidly fatal soon after birth (40). However, as a trade-off, the skewed Th1/Th2 ratio leaves the infant more susceptible to intracellular pathogens such as HIV [reviewed in Ref. (1)]. Interestingly, HIV-exposed but uninfected children show enhanced pro-inflammatory cytokine responses upon stimulation of innate immune cells compared to unexposed children, indicating that altered innate immune ontogeny could contribute to the increased vulnerability of this population to morbidity and poor growth (41).

The intrinsically low level of Th1 responses induced by neonatal innate immune cells is reinforced by the dysregulation and depletion of dendritic cells upon HIV infection [reviewed in Ref. (42)]. Most studies have been undertaken in chronically HIV-infected adults, but perinatally infected children also have reduced frequencies and functionality of dendritic cells and other innate immune cell populations including natural killer (NK) cells (43–45).

Natural killer cells are innate lymphocytes that play a critical role in the control of viral infections through interactions with other host immune cells via the secretion of chemokines and cytokines or by direct cellular cytotoxicity against infected target cells [reviewed in Ref. (46)]. NK cells recognize infected cells either through interaction with antibodies bound to the target cell surface in a process termed antibody-dependent cellular cytotoxicity (ADCC) or through interactions of an intricate network of inhibitory and activating cell surface receptors with ligands on infected cells. For example, NK cells are activated by reduced expression levels of MHC-class-I-molecules on the cell surface of HIV-infected cells via signaling of the inhibitory killer cell Ig-like receptors (KIRs), which is an important complementary mechanism to CD8 T-cell responses that depend on MHC-class-I-recognition [reviewed in Ref. (47)].

Frequencies of neonatal NK cells are similar to adults (48), but there are differences in expression patterns of inhibitory and activating cell surface receptors (49) and reduced cytotoxic activity of cord blood NK cells has been reported consistently (50, 51) [reviewed in Ref. (52)]. NK cells isolated from vertically HIV-infected children have decreased cytolytic activity compared to uninfected children (53) and exhibit reduced ADCC compared to adult cells (54), but neonatal NK cells have been shown to be capable of suppressing replication of CCR5-tropic HIV *in vitro* through a non-cytotoxic chemokine-mediated mechanism (55). Total numbers of NK cells in vertically HIV-infected children decline with age (56) and are only partially reconstituted upon initiation of ART (45). Vertical HIV infection is also associated with increased NK cell activation, differentiation, and loss of perforin expression, which might impair NK cell function and compromise their role in protecting the organism against HIV, co-infections, and cancer (56).

## Developing Immune System: Immune Tolerance and Tregs

The maternal and fetal immune systems are finely adapted toward increased immunological tolerance to avoid uncontrolled damage to the fetus by allogeneic T-cells from the mother on the one hand and pathological reactions to maternal- and self-antigens by the fetus on the other (1). There is immune adaptation away from Th1 and toward Th2 responses during pregnancy (57), although the concept of pregnancy as a generalized state of immunosuppression is no longer supported (58, 59): for example, pregnant women appear to respond well to vaccines during pregnancy (60–62), and even mount anti-fetal CD8+ T-cell response without pregnancy loss (63). The hyporesponsiveness of the fetal adaptive immune system to antigens was previously interpreted as a functional deficit of effector cells, whereas currently the induction of immune tolerance is thought to be an active process that is largely mediated by regulatory T-cells (Tregs) [reviewed in Ref. (64)]. After depletion of CD4+ CD25+ FoxP3+ Tregs, fetal CD4 and CD8 T-cells have been shown to be highly responsive to stimulation *ex vivo* (65). *In utero* exposure to maternal alloantigens induces the development of tolerogenic Tregs that suppress activity of effector T-cells and persist into adulthood (66). The abundant population of Tregs that peaks in the second trimester and gradually declines to adult levels around birth (67) has been shown to originate from fetal hematopoetic stem cells (HSC); these differ from adult HSC based on functional and transcriptional analyses and give rise to a distinct lymphocyte lineage (68).

The role of Tregs in the pathogenesis of HIV infection remains controversial, but several studies have proposed a modulating function in HIV-specific immune responses, chronic immune activation, and inflammation [reviewed in (69)]. Most studies have reported a decrease in absolute numbers of Tregs in chronic HIV infection; however, as the frequency of Tregs is often reported as a percentage of CD4+ T-cells, there appears to be a selective expansion of different Treg subsets within the CD4 population during the CD4 decline that characterizes chronic infection (70). In HIV-infected children, one study found a positive correlation between the proportion of Tregs and HIV viral load, and a negative correlation with CD4 count suggesting a selective expansion of Tregs in pediatric HIV infection (71). Interestingly, in the same study these increased frequencies of Tregs also correlated with the proportion of activated CD8+ T-cells, suggesting the ineffectiveness of these cells to limit immune activation. However, another study in vertically HIV-infected children, showed a depletion of Tregs compared to HIV-negative children when measured as proportion of total T-cells (CD3+ lymphocytes) that was associated with increased levels of immune activation (72). Another study found an association between altered frequencies of Treg subsets and autoantibody production in HIV-positive children highlighting the potential role of Tregs in immunoregulatory pathways that may be disrupted in HIV infection (73). In HIV-exposed but uninfected infants, a significant increase in HIV-specific CD4+ and CD8+ T-cell responses was observed after depletion of Tregs in cord blood mononuclear cells *in vitro* (73, 74) indicating a suppressive function in pathogen-specific adaptive immunity.

## Adaptive Immunity: CD8 T Cells

HIV-specific CD8+ T cells are thought to play a key role in control of viral replication based on studies in humans (75, 76) and SIV-infected monkey models (77, 78). In adults, the rapid decline in viral load during acute infection from several million copies/ml to a viral load set point 3 logs lower is temporally associated with the appearance of HIV-specific CD8+ T-cells (75, 76, 79). Cytotoxic CD8+ T cells interact directly with HIV-infected target cells via the T-cell receptor that recognizes viral peptides presented on the cell surface by MHC-class-I molecules. The HLA-class-I type of an individual affects the rate of disease progression: certain HLA alleles such as HLA-B35 are associated with more rapid progression to AIDS, whereas protective alleles such as HLA-B57 and HLA-B27 are associated with viremic control and slow disease progression (80). One of the main mechanisms proposed for the protective effect of these alleles is their peptide binding specificity, allowing CD8+ T cells to target epitopes located particularly in the Gag region, in which an escape mutation that would evade the immune response is associated with a high cost in viral fitness (81, 82). Transmission of viral variants with an accumulation of costly escape mutations that reduce viral replicative capacity facilitates containment of HIV in the new host and reduces viral load and loss of CD4 cells (83, 84).

In pediatric HIV infection, the HLA type of both mother and child is of relevance for disease progression in the child. In mothers with protective HLA alleles, the virus accumulates immune escape mutations that are associated with a loss of viral fitness, thereby attenuating the transmitted virus, resulting in slower disease progression in the child (85). However, as the child inherits 50% of the HLA alleles from the mother, the virus can be partially pre-adapted to the shared alleles so that the recipient child cannot mount an effective immune response against the escaped epitopes and fails to contain viral replication (86). The beneficial effect of protective HLA alleles is therefore greatest if they are not shared between mother and child, and the child can employ them to mount effective immune responses (85). In a study of vertically HIV-infected children and adolescents, the protective allele HLA-B57 was overrepresented in the group of children with slow disease progression (87). In a recent genomic study of over 1000 vertically HIV-infected children, the protective HLA alleles HLA-B57, -B27, -B14, -Cw8, and -DRB1*10 were the SNPs most strongly associated with low baseline HIV-RNA levels (35).

The adaptive immune system starts to develop as early as 7–9 weeks of gestation when T-cell progenitor cells populate the thymus (88). In vertically HIV-infected children, HIV-specific CD8+ T cell responses can be detected from birth (89, 90), but less frequently and at a lower magnitude than in older children or adults (90–92). However, these responses seem to be insufficient to control viremia, as there is typically no rapid decline in viral load in pediatric HIV infection as opposed to the decline in viral load after acute infection in adults [see above and Ref. (25)]. In infants, neither breadth nor magnitude of HIV-specific CD8 T-cell responses at 1 month of age correlate with viremic control or survival at 12 months (93), but Gag-specific CD8 T cell responses are higher in infants who survive to 1 year of age (94). The magnitude and breadth of Gag-specific CD8 T cell responses correlated negatively with viral load in one study (95), but not in another (96). Also, in a study of older children and adolescents who were categorized as progressors and non-progressors, there was no difference in magnitude and breadth of HIV-specific CD8 T cell responses between the two groups (87). However, magnitude and breadth of HIV-specific CD8 responses increase with age (90, 92, 96).

Broad Gag-specific CD8+ T cell responses have consistently been shown to be associated with reduced viral load (97, 98) in HIV-infected adults. But whereas Gag is the most immunogenic HIV-protein in adults, infants preferentially target epitopes in the more variable proteins Env and Nef (92, 95) with an increasing proportion of Gag-specific responses only later in life (95).

In addition to the specificity and magnitude, the effectiveness of a CD8 T cell response to control viral replication is also determined by its quality as measured by the ability to degranulate and produce different effector cytokines simultaneously (99, 100). The frequency of HIV-specific CD8 T cell that exhibit three different effector functions or more (CD107+, IL-2+, IFN-gamma+) is reduced in children younger than 2 years but increases with age (95). In another study, the functional profile of HIV-specific CD8 T cell responses predicted the rate of subsequent disease progression in perinatally HIV-infected infants showing that more polyfunctional responses were associated with slower disease progression (101).

Other functional measures that have been described for effective CD8 T cell responses include the proliferative capacity of a T cell clone upon antigen stimulation *in vitro* (102). In children, the proliferative capacity of HIV-specific CD8 T cells has thus far not been studied in detail and published data are only available for CD4 T cells at present.

## Adaptive Immunity: CD4 T cells

To maintain fully functional CD8+ T cell responses, the help of CD4+ T cells with the same antigen-specificity is required (103, 104). In the mouse model, depletion of CD4 T helper cells during acute infection results in ineffective CD8 T cell memory responses (105, 106). CD4+ T cells are the major target for HIV and massive depletion of CD4+ T cells results during acute infection in adults (107, 108), with ongoing preferential infection of activated and HIV-specific CD4 T cells in chronic infection (109). This depletion of HIV-specific CD4+ T helper cells has been proposed as a main mechanism for failure to control HIV successfully, given their central role in orchestrating diverse cellular and humoral immune functions (110).

In addition to providing help to CD8 T cells (111), HIV-specific CD4 T cells have been shown to have intrinsic direct cytotoxic activity against infected cells (112–114). Increased breadth, especially of Gag-specific CD4 T cell responses, is inversely correlated to viral load in chronically infected adults; elite or viremic controllers show a higher ratio of Gag- vs. Env-specific CD4 responses (115). Another study in a large cohort of ART-naïve HIV-1-clade-C infected adults also found that the magnitude of Gag-specific CD4 T cell responses was associated with higher CD4 counts and lower viral loads (116).

As discussed above in relation to innate immunity, there is a selective impairment of CD4 T cell responses in early childhood with a shift from Th1 toward Th2 type responses (117, 118) and poor generation of persistent Th1 memory responses (119). This has been attributed to low production of Interleukin-12 (120), which is required for Th1-polarization and maintenance of Th1 effector functions (121, 122), but does not reach adult levels until adolescence. The bias toward Th2 responses in early life is reinforced by the increased capacity of neonatal monocytes and dendritic cells to produce Interleukin-10, an immunomodulatory cytokine that induces a shift of T cell induction toward Th2 and Th17 profiles (38).

During fetal development in non-pathologic sterile conditions, the T cell compartment is considered to be mostly naïve and tolerogenic, although a recent study highlighted the existence of CD4 T cell effector memory subsets with Th1, Th2, and Th17 functional profiles in cord blood of healthy newborn infants (123). HIV-specific CD4 T cell responses can be primed *in utero* (124), but are detected in infants at significantly lower frequencies than in chronically infected adults and increase with age (92, 95). These low levels of HIV-specific Th1 responses in early life coincide with the Th1/Th2 bias and the expanded population of suppressive regulatory T cells as described above. In the macaque model, SIV-specific CD4 T cell responses are suppressed in infants by regulatory T cells that show greater *in vitro* suppressive activity and are present at higher frequencies than in adults and have been associated with impaired immune control (125).

Gag-specific CD4 T cell responses were detectable at higher frequencies at 3 months of age in vertically HIV-infected children who survived to 12 months, compared to those who died, and Gag-specific responses at 3 and 6 months of age were negatively correlated with VL (94). Another study detected Gag-specific CD4 responses only in a minority of older vertically infected children (median age 9.9 years). Those with detectable responses had lower viral loads than non-responders and the magnitude of the response was inversely correlated with viral load; no association was observed between CD8 T cell responses and viral load (96).

The functional capability of CD4 T cells to produce IL-2 is causally linked to the proliferative capacity of both CD4 and CD8 T cell subsets, a parameter consistently correlated with immune control and slow disease progression [reviewed in Ref. (126)]. HIV-specific proliferative responses of CD4 T cells are detected only in a fraction of viremic chronically infected adults and are inversely correlated with VL if present (127, 128); IL-2 producing HIV-specific CD4 responses are associated with slow disease progression in adults (129, 130). Interleukin-2 is also important for the differentiation, activation, and proliferation of NK cells, Th1 and Th2 CD4 T cells, B cells, Tregs, and memory CD8 T cells, highlighting the key immunoregulatory role of these CD4 T cells in the immune response (131).

The proliferative capacity of HIV-specific CD4 T cells is selectively impaired in vertically infected children with uncontrolled viral replication (124, 132), but, in contrast to adult infection, can be rescued with administration of ART (133). The magnitude of the response increases with age and is strongest in children maintaining viral suppression on ART (133). Interestingly, proliferative and IL-2 producing CD4 responses were also detected in HIV-exposed uninfected infants but were mostly absent in infected children (134). Polyfunctional HIV-specific CD4 T cell responses, especially Interleukin-2 producing cells, were less frequently detected in younger children, but if present, they were associated with low viral loads and remarkably slow disease progression (95). However, it remains unclear whether these responses actually mediate viral control or are simply a proxy for undisrupted immunoregulatory networks.

## Immune Exhaustion

Chronic HIV infection and persistent antigenic stimulation lead to more terminally differentiated T cell populations with loss of effector functions and proliferative capacity upon antigenic stimulation [reviewed in Ref. (3)]. This state of “Immune Exhaustion” is characterized by the upregulation of inhibitory co-receptors on CD4 and CD8 T cells such as PD-1 (135, 136), Tim-3 (137), CD160 (138), LAG-3 (139), and CTLA-4 (140) that correlate with markers of disease progression in chronic HIV infection (136, 137, 139–141). As immune functions can be partially restored upon antibody-mediated blockade of these receptors (136, 137, 139, 140), they present an attractive target for immunotherapeutic intervention in HIV infection, although immune-related adverse events remain a concern (142, 143).

In pediatric subjects, few studies have been conducted to elucidate the role of immune exhaustion in chronic HIV infection. One study found an expansion of Tim-3+ CD4 and CD8 T cells in vertically HIV-infected adolescents and a correlation of Tim-3 expression on CD8 T cells with viral load (144). In the same study, PD-1 expression only correlated with the frequency of activated CD8 T cells. This is consistent with another report that described a correlation of PD-1 expression on CD8 T cells with immune activation markers and the magnitude of HIV-specific CD8 T cell responses, but not with viral load, suggesting that immune exhaustion is driven more by chronic immune activation than directly by viral replication (96).

## Immune Activation

Persistent systemic immune activation plays a key role in the pathogenesis of HIV infection and is regarded as the driving force in generalized immune dysregulation, chronic inflammation (145, 146), depletion of CD4 T cells (147, 148), and progression to AIDS (149) [reviewed in (4)]. Recent studies suggest that much of the CD4 depletion is caused by a process called “pyroptosis,” whereby abortive HIV infection of CD4+ T cells causes a highly inflammatory form of cell death, which drives accumulation of further CD4 cells and an ongoing cycle of inflammation and cell death (150, 151). T cell immune activation, as commonly measured by expression of activation markers such as HLA-DR and CD38 on CD4 and CD8 T cells, is a stronger predictor of disease progression than viral load in adults (152). Similarly, in vertically HIV-infected infants, the subsequent rate of disease progression can be predicted by T cell activation levels at 1–2 months of age (153). Both CD4 and CD8 immune activation show a strong inverse correlation with CD4 percentage, but interestingly no correlation is observed between immune activation and viral load in children (72, 154). In vertically HIV-infected children who maintain high CD4 counts without ART (often referred to as pediatric slow progressors), immune activation levels are strikingly low despite high viral loads (Muenchhoff et al., unpublished data). This resembles the phenotype of non-pathogenic infection in the natural hosts of SIV, such as African Green Monkeys and Sooty Mangabeys (155), in whom CD4 counts are maintained despite high levels of viremia, due to attenuated immune activation (156). In acute infection, sooty mangabeys also show high levels of immune activation and a robust innate immune response that is associated with a generalized upregulation of Interferon Stimulated Genes (ISGs, see innate immunity), but this initial response is rapidly resolved, suggesting the involvement of active immunoregulatory mechanisms that dampen detrimental excessive immune activation (157).

After initiation of ART, immune activation levels in children and adults decrease (154) but usually remain elevated compared to HIV-negative controls and this residual immune activation despite ART is associated with incomplete immune reconstitution, increased non-AIDS comorbidities, and mortality (158–160) [reviewed in Ref. (161)]. Persistent immune activation is associated with increased levels of pro-inflammatory cytokines and elevated coagulation markers that have been linked to cardiovascular disease and cancer in adults [reviewed in Ref. (4, 162)], emphasizing a potential role for adjunctive immunomodulatory interventions together with ART to reduce residual immune activation in people living with HIV.

The cause of systemic immune activation in HIV infection has not yet been fully elucidated but appears to arise from a complex interplay between HIV and the host involving several molecular and cellular mechanisms including innate and adaptive immune responses that create a pro-inflammatory milieu, fibrosis of lymphoid tissue, uncontrolled co-infections, and breach of the intestinal barrier resulting in translocation of microbial products into the systemic circulation [reviewed in Ref. (161)].

## Microbial Translocation

From early stages of HIV infection, the gastrointestinal tract is a target for HIV-induced pathology, with severe depletion of mucosal CD4 T cells (in particular Th17 cells), dendritic cells, and innate lymphoid cells (107, 163, 164) disrupting mucosal immunity and the intestinal epithelial architecture [reviewed in (2, 165)]. This breach in the mucosal barrier results in translocation of microbial products from the gut into the systemic circulation leading to immune activation (166). A critical difference between non-pathogenic SIV infection (sooty mangabeys, African green monkeys) and pathogenic infection (rhesus macaques, pig-tailed macaques) is in preservation of CD4+ Th17 cells in the sooty mangabeys and African Green monkeys (167). Markers that are used to measure microbial translocation include direct microbial products like lipopolysaccharide (LPS), a component of the Gram-negative bacterial cell wall, and conserved bacterial ribosomal 16sRNA, or indirect markers such as sCD14 as a soluble marker of monocyte activation (168).

The immaturity of the gut in early life, characterized by increased epithelial permeability (169) and the evolution of the gut microbiota following bacterial and viral colonization (170) might influence the degree of microbial translocation in pediatric populations. Plasma levels of LPS in uninfected children initially increase after birth to reach a plateau at about 6 months of age (171) and then decrease again after the age of 2 years (172).

In vertically HIV-infected infants, levels of microbial translocation are elevated compared to uninfected controls and persist at a higher level after initiation of ART (173). Persistent microbial translocation correlates with cellular and soluble markers of immune activation in children on ART suggesting an important role in immune reconstitution (174). In a recent study in untreated HIV-infected children, microbial translocation was strongly correlated with monocyte and T cell activation and PD-1 expression levels on CD4 and CD8 T cells demonstrating the connection between microbial translocation, immune activation, and immune exhaustion (175).

## Adaptive Immunity: B Cells and Antibodies

HIV-specific antibodies have been in the focus of intense research for several decades in the quest for a prophylactic HIV vaccine. Recently, hope has been fueled by the results of the RV144 vaccine trial, which showed modest vaccine efficacy (176) and identified antibodies against the V1V2 HIV-envelope region as a correlate of protection in vaccinees (177). Antibodies can mediate antiviral activities by binding either to free virus or to viral particles on infected cells. The Fc-region of the bound antibody can mediate phagocytosis of the virus or infected cells, activation of innate immune cells resulting in ADCC against infected cells (see innate immunity), or activation of the complement system leading to opsonization and lysis of the virus or infected cells [reviewed in Ref. (178)]. An important function of antibodies especially in regard to sterilizing immunity is the ability to neutralize the virus by binding to the viral surface and inhibit cellular infection. The surface of HIV consists of the gp41 trans-membrane protein and the heavily glycosylated gp120 surface protein, which are both highly variable between different viral variants. Therefore, antibodies elicited by an effective protective vaccine would need to be broadly neutralizing across different viral subtypes (179).

These broadly neutralizing antibodies (bNABs) have been shown to occur naturally in a minority (20%) of HIV-infected subjects over the course of several years post-infection (180–182) during a process of intense somatic hypermutation (183). A recent study identified several young children with HIV-specific antibodies that had surprisingly high neutralizing breadth (184). Interestingly, the proportion of children with high neutralization scores was greater than in large-scale cohort studies of chronically HIV-infected adults and high levels of neutralizing breadth were already reached at an age of ~2 years, suggesting accelerated affinity maturation. These findings were especially surprising as it had previously been shown that infants produce lower antibody titers with less diversified somatic mutation upon infection and vaccination shortly after birth [reviewed in Ref. (185)]. As higher set point, viral load levels are associated with increased emergence of bNABs in adults (186) and in these young children (184), it has been hypothesized that the high antigenic load in pediatric HIV infection boosts development of NAB breadth. Also other factors like the shift toward Th2 responses in early life (as discussed above) could promote B-cell function and somatic hypermutation. Differential proportions of IgG-subclasses in children with a preponderance of IgG1 and IgG3 (185) that have higher neutralizing potency (187, 188) could also contribute to this finding. CD4+ T follicular helper (Tfh) cells are thought to be crucial in providing help to B cells in the germinal center to support antibody maturation [reviewed in Ref. (110)], but there have been no studies of Tfh cells in HIV-infected children to date. Further investigation in this field could yield important findings for vaccine development.

## Mississippi Child/Reservoir/Cure

The case report of an *in utero* HIV-infected child who appeared to have been “functionally cured” after early initiation of ART was received with much excitement. The child initiated ART within 30 h of life and stayed on ART until 18 months of age when ART was discontinued by the mother. In subsequent visits to the clinic half a year later and in the successive follow-up period, viral load levels remained undetectable in the absence of ART and no signs of ongoing viral replication or HIV-specific T-cell or antibody responses were detected (5), hence the child was labeled “functionally cured.” However, after more than 2 years of follow up with undetectable HIV-RNA levels after treatment cessation, there was a recent report of rebound viremia in this child.

The main obstacle to HIV eradication is a pool of long-lasting, treatment-resistant viral reservoirs that only decay slowly on ART and are a source for viral rebound after treatment cessation [reviewed in Ref. (189, 190)]. Different cell types and body compartments are thought to contribute to the viral reservoir, but major sources of replication-competent pro-viral DNA during effective ART are resting central and transitional memory CD4+ T cells (191). In this regard, children might be partially protected against the early establishment of viral reservoirs in these cell populations, because neonates have much lower frequencies of CD4 T cells of central memory phenotype than adults (22, 23).

The frequency of latently infected CD4 T cells in children strongly depends on early initiation of ART and the time to achieve virologic suppression (192). A cross-sectional study found low to undetectable levels of integrated pro-viral DNA in CD4 cells of children starting treatment before 6 months of age after more than 3 years of follow-up (193). A recent longitudinal study compared perinatally infected youth initiating treatment before or after 3 months of age (194). In contrast to the late treatment group, all children starting ART before 3 months had undetectable HIV-RNA plasma levels using highly sensitive assays but integrated pro-viral DNA could be detected by PCR in all subjects even after follow-up periods of up to 17 years. In the early-treated group, transitional memory CD4 T cells had a larger contribution to the pool of infected cells than longer lasting central memory CD4 T cells. These findings are consistent with a study of adult post-treatment controllers who initiated ART during acute HIV infection (195), indicating that early treatment can prevent seeding of long-lived cellular reservoirs. It has to be noted that only a fraction of integrated viral DNA codes for replication-competent virus (196) and so the standard assay to measure the size of the functional latent viral reservoir at present is a limiting dilution viral outgrowth assay (197, 198). Using this assay, only one of the early-treated children in this study had inducible replication-competent virus, as opposed to all children receiving ART at a later stage, although limited sample availability in pediatric studies reduces the sensitivity of these assays. Caution also has to be taken as rebound viremia is observed even in patients with extraordinarily low viral burden in the peripheral blood compartment (199) and a large proportion of the viral reservoir is actually located within the gut and other tissues that are not accessed in most studies (200). Reliable biomarkers to predict successful drug-free remission upon treatment cessation are therefore needed.

Apart form timing of ART initiation, the size and constitution of the viral reservoir is affected by host immunological factors including persistent immune activation that fuels replenishment of the reservoir during ART [reviewed in Ref. (161)] and effective immune responses that can reduce the pool of infected memory cells (201, 202). To achieve a cure of HIV in a broader group of patients will therefore most likely require a multifaceted approach that could include induction of potent HIV-specific immunity prior to reversion of HIV-latency in combination with immunomodulatory interventions.

## Conclusion

In summary, immunity to HIV in early life differs from that in adulthood not only in quantity but also in quality. This results from the adaptations of the immune system to the abundance of antigenic stimulation and pathogen exposure *in utero* and after birth. The initial ontogeny of the immune system is characterized by a more tolerogenic state of innate and adaptive immunity that prevents potentially harmful autoimmunity and inflammatory responses, and is geared toward preferential protection against extracellular pathogens. As a consequence, vertically acquired HIV infection is poorly controlled and marked by rapid progression to AIDS and death without effective interventions. Chronic infection results in systemic immune activation that drives immunopathology with functional immune exhaustion, increased susceptibility to co-infections, and inflammatory comorbidities. Coverage rates of PMTCT, early infant diagnosis, and pediatric ART need to be improved worldwide to reduce the global disease burden among children. For those children who become infected despite PMTCT, novel therapeutic strategies need to be developed targeting persistent immune dysregulation and the viral reservoir to potentially achieve drug-free remission.

## Conflict of Interest Statement

The authors declare that the research was conducted in the absence of any commercial or financial relationships that could be construed as a potential conflict of interest.
